# Long-term neurological outcomes of offspring misdiagnosed with fetal growth restriction

**DOI:** 10.1007/s00404-024-07525-y

**Published:** 2024-05-01

**Authors:** Amir Snir, Omri Zamstein, Tamar Wainstock, Eyal Sheiner

**Affiliations:** 1 The Obstetrics and Gynecology Division, Soroka University Medical Center, Ben-Gurion University of the Negev, POB 151, Beer-Sheva, IL Israel; 2 The Department of Public Health, Faculty of Health Sciences, Ben-Gurion University of the Negev, Beer-Sheva, Israel

**Keywords:** False FGR diagnosis, Long-term neurological outcomes, Preterm labor, Early term labor

## Abstract

**Objective:**

Fetal growth restriction (FGR) is a major determinant of adverse short- and long-term perinatal outcomes. The current definition of FGR (estimated fetal weight measurement < 10th percentile) may lead, at times, to a false diagnosis of fetuses that are eventually born appropriate for gestational age (AGA). Our objective was to investigate the potential association between a misdiagnosis of antepartum fetal growth restriction and long-term neurological outcomes in offspring.

**Study design:**

A population-based cohort analysis was performed including deliveries between the years 1991–2020 in a tertiary medical center. We compared neurological hospitalization during childhood among AGA infants falsely diagnosed as FGR versus AGA infants without a false FGR diagnosis. A Kaplan–Meier survival curve was used to assess cumulative morbidity and a Cox proportional hazards model was employed to control for confounders.

**Results:**

During the study period, 324,620 AGA infants met the inclusion criteria; 3249 of them were falsely classified as FGR. These offspring had higher rates of hospitalizations due to various neurological conditions, as compared to those without an FGR diagnosis (OR 1.431, 95% CI 1.278–1.608; P < 0.001). In addition, cumulative hospitalization incidence was elevated in the FGR group (log-rank P-value < 0.001). When controlling for confounders, a false FGR diagnosis remained independently associated with long-term neurological morbidities (adjusted HR 1.086, 95% CI 1.003–1.177, P = 0.043).

**Conclusion:**

Misdiagnosis of FGR in the antepartum period is associated with an increased risk for offspring long-term neurological morbidities.

## What does this study add to the clinical work

**Table:** 

Our research indicates a link between incorrect diagnoses of fetal growth restriction (FGR) and adverse long-term neurological outcomes. Improving the accuracy in diagnosing FGR could potentially lessen related complications.	

## Introduction

Fetal growth restriction (FGR) is a prevalent obstetric condition wherein the fetus fails to achieve its growth potential due to an underlying pathological factor [1]. FGR is typically defined as an estimated fetal weight (EFW) below the 10th percentile for gestational age [2], occasionally accompanied by abnormal Doppler blood flow patterns [3].

FGR is associated with an increased risk of short-term adverse perinatal outcomes such as hypoglycemia and prematurity [4], in addition to long-term morbidities such as neurodevelopment abnormalities [5], respiratory [6], and cardiovascular morbidity [7]. Key factors contributing to FGR include maternal conditions, placental abnormalities, and genetic factors [8]. However, the diagnosis of FGR is far from straightforward and continues to raise challenges. The complexity of accurately identifying true cases of FGR is compounded by the prevalence of false positives, where healthy fetuses are misclassified as growth-restricted. Serial ultra-sonograms are important for the evaluation of FGR, and umbilical artery Doppler velocimetry is used to guide pregnancy management decisions [9].

Unfortunately, the current most common clinical mentioned definition for FGR is inaccurate and may lead at times to falsely diagnosed fetuses that are eventually born appropriate for gestational age (AGA). The phenomenon of false FGR diagnosis seems more common than generally perceived. Monier et al. found that approximately 50% of the neonates diagnosed with FGR in the antepartum period, were eventually born AGA, i.e., above the 10th weight percentile [10]. This is concordant with a previous study, which also documented low detection rates for FGR [11].

Accurate diagnosis of FGR is crucial because it helps to take informed decisions about patient and pregnancy care. False FGR diagnoses can lead to unnecessary interventions such as labor inductions, cesarean deliveries (CD), or premature birth, which can pose additional risks to both the pregnant patients and the fetus. Moreover, the psychological distress caused by a false FGR diagnosis can be substantial, causing anxiety and fear for the expectant individuals.

Previous studies have associated a false FGR diagnosis with short-term adverse perinatal outcomes [12]. This study seeks to investigate the long-term neurological outcomes of offspring in cases where pregnancies were initially misdiagnosed with FGR but ultimately resulted in the delivery of appropriately grown neonates.

## Materials and methods

A population-based cohort study was performed comparing the risk for long-term neurological diagnoses according to whether the newborns were falsely diagnosed with FGR or not. This cohort analysis included all singleton deliveries with a birth weight appropriate for gestational age (AGA) which occurred during the years of 1991 to 2020. Missing gestational age, fetuses with congenital malformations, and cases of perinatal deaths were excluded.

The study was conducted at the Soroka University Medical Center (SUMC). This center is the only hospital in southern Israel, serving the entire population of the region totaling over one million inhabitants, thus the study is based on nonselective population data. The study is approved by the institutional review board (in accordance with the Helsinki Declaration). Neurological disorders in accordance with International Classification of Diseases, Ninth Revision (ICD-9) were compared among AGA offspring with and without a false diagnosis of FGR. Follow-up was conducted up to the age of 18 years. The relevant neurological diagnoses were given based on an evaluation performed on offspring’s admission to the hospital or based on the patient’s history. It may have been the main reason for admission or a background disorder. For diagnosing FGR, we used Dollberg and colleagues’ birth weight growth curves. These curves are a commonly employed, population-based standard for evaluating weight percentiles based on the birth weights of live-born infants in Israel [13]. Fetal weights were estimated using sonographic measurements. After birth, we applied these same growth curves to classify the neonate’s weight. When necessary, the timing of delivery was determined based on several factors: gestational age, the severity of suspected FGR, fetal heart rate monitoring, the biophysical profile, and Doppler velocimetry results.

Data were collected from the computerized hospitalization database of SUMC (“Demog-ICD9”) and the computerized perinatal database of the obstetrics and gynecology department. The Demog-ICD9 database includes demographic information and ICD-9 codes for all medical diagnoses made during encounters with SUMC. In order to ensure maximal integrity and accuracy, experienced medical secretaries routinely review the information before entering it into the database. Coding is performed after assessing medical and perinatal records as well as routine hospital documents.

Statistical analysis was performed using SPSS (version 24) and STATA (version 12.0) software. Differences between the groups were assessed using χ^2^ test, *t* test, in accordance with the variable type and its distribution. Kaplan–Meier survival curves were used to compare cumulative hospitalization incidences over time, and the differences were analyzed using the log-rank test. To establish an association with cumulative hospitalization incidence, while controlling for potential confounders, we used a multivariate Cox proportional hazards model to account for repeated occurrence of mothers and the dependence among siblings in the cohort. *P*-values < 0.05 were considered statistically significant.

## Results

During the study period, 324,620 AGA infants met the inclusion criteria. 3249 of them (1%) were falsely classified as FGR in the antepartum period. Table 1 demonstrates the maternal and obstetrical outcomes, according to false FGR diagnosis, or not. Pregnancies with false FGR diagnosis demonstrated higher rates of hypertensive disorders (14.7% vs. 4.3% *p* < 0.001), preterm delivery (36.4% vs. 6.6%, *p* < 0.001), and CD (33.9% vs. 13.0%, *p* < 0.001). The fetal weights in the false FGR group followed a normal distribution but were consistently lower than those of the non-false FGR newborns across all percentiles. The mean gestational age for the group falsely diagnosed with FGR was 36.7 ± 2.47.

**Table 1:** Maternal and obstetrical outcomes, according to false FGR diagnosis

Characteristic		FGR misdiagnosis (n = 3249)	No FGR misdiagnosis (n = 321,371)	Odds Ratio	P value
Hypertensive disorders (%)		14.7	4.3	3.82	< 0.001
Oligohydramnios (%)		13.3	1.9	7.94	< 0.001
Obesity (%)		1	1.1	0.89	0.532
Smoking (%)		1.7	0.7	2.45	< 0.001
Nulliparity (%)		36.5	24.0		< 0.001
Ethnicity (%)	Bedouin	45.8	54.2	1.40	< 0.001
	Jewish	54.2	45.8	1.40	< 0.001
Maternal age (Mean ± SD)		27.5 ± 5.9	28.2 ± 5.8		< 0.001
Labor induction (%)		41.2	20.8	2.67	< 0.001
Non-reassuring fetal heart rate (%)		9.1	5.0	1.90	< 0.001
Cesarean delivery (%)		33.9	13.0	3.43	< 0.001
Preterm delivery (< 34 weeks) (%)		9.2	1.4	6.99	< 0.001
Preterm delivery (< 37 weeks) (%)		36.4	6.6	8.11	< 0.001
Low-birth weight (≤ 2500 g) (%)		58.9	4.3	31.71	< 0.001
Very low birthweight (≤ 1500 g) (%)		6.2	0.6	11.13	< 0.001

These infants exhibited a higher rate of long-term neurological morbidities as compared to those without a false FGR diagnosis (OR 1.431, 95% CI 1.278–1.608; *p* < 0.001, Table 2). Specifically, movement disorders (4.2% vs. 2.9%, *p* < 0.01), cerebral palsy (0.4% vs. 0.2%, *p* < 0.002), and developmental disorders (2.2% vs. 0.8%, *p* < 0.001) were each significantly more common in the false FGR diagnosis group.

**Table 2:** Long term neurological outcomes of offspring according to false diagnosis of FGR

	FGR misdiagnosis (n = 3249)	No FGR misdiagnosis (n = 321,371)	OR	95% CI	P value
Autism spectrum disorder	2 (0.1%)	207 (0.1%)	0.956	0.237–3.848	0.949
Eating disorder	30 (0.9%)	1331 (0.4%)	2.241	0.1558–3.223	< 0.001
Sleep disorders	2 (0.1%)	585 (0.2%)	0.676	0.253–1.808	0.432
Movements disorders	138 (4.2%)	9464 (2.9%)	1.462	1.231–1.736	< 0.001
Cerebral palsy	14 (0.4%)	609 (0.2%)	2.279	1.340–3.876	< 0.002
Mental health disorders	101 (3.1%)	9104 (2.8%)	1.100	0.902–1.343	0.346
Attention deficit hyperactivity disorder	6 (0.2%)	309 (0.1%)	1.922	0.856–4.315	0.107
Developmental disorders	70 (2.2%)	2649 (0.8%)	2.649	2.084–3.368	< 0.001
Degenerative or demyelization neurological conditions	8 (0.2%)	603 (0.2%)	1.313	0.653–2.640	0.443
Headache	4 (0.1%)	91 (0.0%)	4.352	1.598–11.852	< 0.002
Total neurologic-related morbidity	330 (10.2%)	23,489 (7.3%)	1.434	1.278–1.608	< 0.001

**Fig. 1:**
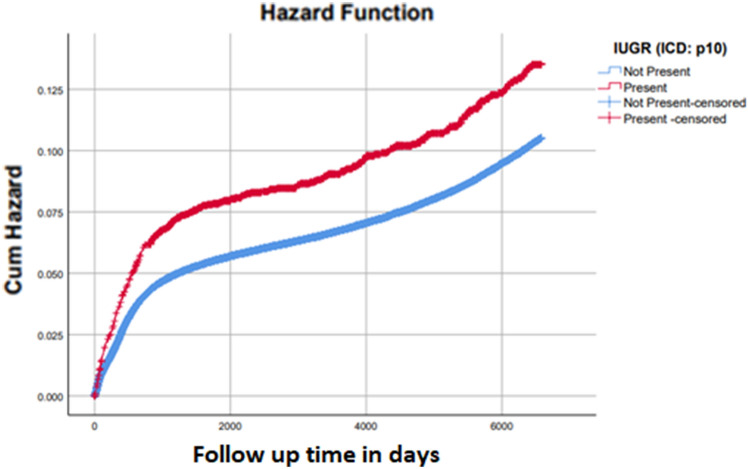
Cumulative incidence of hospitalization with neurological morbidity in offspring according to whether falsely classified as FGR or not (Log Rank P value < 0.001)

The cumulative incidence of long-term neurological morbidity was significantly higher among false FGR diagnosis (Kaplan–Meier survival curve Log rank *p* < 0.001, Fig. 1). In the Cox proportional hazard regression model, an independent association was found between false FGR diagnosis and long-term neurological morbidity, after adjusting for gestational age, birth year (since our study included a long range of years), CD and hypertensive disorders as possible confounders (adjusted HR 1.086, 95% CI 1.003–1.177, P = 0.043).

## Discussion

Based on the findings of our study, it has been observed that fetuses suspected to be growth-restricted who subsequently had a normal birth weight were at an increased risk of experiencing long-term neurological morbidity during childhood. This includes a heightened likelihood of conditions such as movement disorders, developmental impairments, and cerebral palsy.

Prior research on this subject has shown that false diagnoses of FGR have been linked to higher rates of early-term deliveries, preterm CD, and increased risk for short-term adverse neonatal outcomes. Consistent with our findings, a study assessing the consequences of misdiagnosed FGR on the mode of delivery indicated a tenfold increase in the rates of preterm CD (12.7% versus 1.2%) [14]. Gabbay-Benziv et al., additionally found that these offspring were at increased risk for transient tachypnea of the newborn, mechanical ventilation, hypoglycemia, and NICU admissions [12].

Our study specifically focuses on the less-explored area of long-term neurological impacts resulting from a misdiagnosed FGR. Analyzing our results, we suggest a possible explanation for the difference in the observed outcomes. The elevated prevalence of long-term neurological complications may be connected to iatrogenic early deliveries, as physicians might be inclined to induce preterm or early-term labor and perform CD in cases of suspected FGR, potentially overlooking the iatrogenic risks [10]. Preterm birth is an established risk factor associated with long-term adverse neurological outcomes in offspring. The immature brain is susceptible to both the primary brain damage (such as white matter injury, germinal intraventricular hemorrhage, and cerebellar hemorrhage) and the secondary consequences of incomplete maturation [15]. Furthermore, there is also evidence to suggest that even early-term deliveries are associated with long-term adverse outcomes [16–19] specifically neurodevelopmental impairment compared with full-term birth [20]. In a study conducted by Baumfeld et al., it was demonstrated that CD, in contrast to vaginal delivery, emerged as an independent risk factor for pediatric neurological hospitalizations [21]. Notably, the results in our study retained their significance even after accounting for confounders such as gestational age at birth, hypertensive disorders, CD, and birth year. A possible reason for the increased morbidity is that even though these fetuses reached the “AGA threshold,” they might have still experienced restricted growth in utero. These infants could potentially have a similarly unfavorable prognosis as those with lower birth weights [22].

This interpretation aligns with our observation that the fetal birth weights in the false FGR group were consistently smaller than those in the non-false FGR group across all percentiles, despite a normal distribution. However, this difference might also be attributed to an earlier gestational age, considering that all misclassified cases in the group were born AGA and that gestational age was accounted for in the multivariable analysis.

Our study has several limitations. First, it only includes diagnoses of hospitalized children, potentially excluding those with mild-to-moderate neurological issues not requiring hospitalization. A potential solution is a follow-up study integrating community medical records with hospital data. Secondly, retrospective studies may have coding errors, despite skilled review. Thirdly, immigration and potential loss to follow-up are considerations, but their effects likely apply similarly to both groups.

Our study’s major strength stems from the fact that SUMC is the sole tertiary hospital in the southern region of Israel. That, combined with free essential health insurance provided to each citizen of Israel, makes it safe to assume that if a woman gave birth to a child in SUMC, the child would reach SUMC when in need of major medical assistance.

In conclusion, our study shows an independent association between misdiagnosed FGR and an elevated risk of long-term neurological health issues in offspring. It is possible that these increased neurological morbidities may be a consequence of iatrogenic deliveries at a lower gestational age and prompt the question of the adequacy of ultrasound measurements for FGR detection. Additional research may help develop a more effective method for predicting FGR, particularly in the third trimester, through wider application of an individualized growth curve and the implementation of routine third-trimester screening for FGR [23, 24].

## Data Availability

The data supporting this article can be provided upon a reasonable request to the corresponding author, subject to institutional review board approval and conditions.

## References

[1] Melamed N, Baschat A, Yinon Y, Athanasiadis A, Mecacci F, Figueras F, Berghella V, Nazareth A, Tahlak M, McIntyre HD, Da Silva Costa F, Kihara AB, Hadar E, McAuliffe F, Hanson M, Ma RC, Gooden R, Sheiner E, Kapur A, Hod M (2021) FIGO (International federation of gynecology and obstetrics) initiative on fetal growth: Best practice advice for screening, diagnosis, and management of fetal growth restriction. Int J Gynecol Obstet 152(S1):3–57. 10.1002/ijgo.13522 10.1002/ijgo.13522 PMC8252743 33740264

[2] Sharma D, Shastri S, Farahbakhsh N, Sharma P (2016) Intrauterine growth restriction—part 1. J Matern Fetal Neonatal Med 29(24):3977–3987. 10.3109/14767058.2016.1152249 26856409 10.3109/14767058.2016.1152249

[3] Unterscheider J, Daly S, Geary MP, Kennelly MM, McAuliffe FM, O’Donoghue K, Hunter A, Morrison JJ, Burke G, Dicker P, Tully EC, Malone FD (2013) Optimizing the definition of intrauterine growth restriction: the multicenter prospective PORTO Study. Am J Obstet Gynecol 208(4):290.e1-290.e6. 10.1016/j.ajog.2013.02.007 23531326 10.1016/j.ajog.2013.02.007

[4] Regev RH, Lusky A, Dolfin T, Litmanovitz I, Arnon S, Reichman B (2003) Excess mortality and morbidity among small-for-gestational-age premature infants: a population-based study. J Pediatr 143(2):186–191. 10.1067/s0022-3476(03)00181-1 12970630 10.1067/S0022-3476(03)00181-1

[5] Sacchi C, Marino C, Nosarti C, Vieno A, Visentin S, Simonelli A (2020) Association of intrauterine growth restriction and small for gestational age status with childhood cognitive outcomes. JAMA Pediatr 174(8):772. 10.1001/jamapediatrics.2020.1097 32453414 10.1001/jamapediatrics.2020.1097 PMC7251506

[6] Peles G, Paz-Levy D, Wainstock T, Goldbart A, Kluwgant D, Sheiner E (2022) Pediatric respiratory hospitalizations in small for gestational age neonates born at term. Pediatr Pulmonol 57(3):754–760. 10.1002/ppul.25797 34931470 10.1002/ppul.25797

[7] Neimark E, Wainstock T, Sheiner E, Fischer L, Pariente G (2019) Long-term cardiovascular hospitalizations of small for gestational age (SGA) offspring born to women with and without gestational diabetes mellitus (GDM). Gynecol Endocrinol 35(6):518–524. 10.1080/09513590.2018.1541233 30626227 10.1080/09513590.2018.1541233

[8] Albu AR, Anca AF, Horhoianu VV, Horhoianu IA (2014) Predictive factors for intrauterine growth restriction. J Med Life 7(2):165–171 25408721 PMC4197512

[9] Aditya I, Tat V, Sawana A, Mohamed A, Tuffner R, Mondal T (2016) Use of Doppler velocimetry in diagnosis and prognosis of intrauterine growth restriction (IUGR): a review. J Neonatal-Perinat Med 9(2):117–126. 10.3233/npm-16915132 10.3233/NPM-16915132 27197939

[10] Monier I, Blondel B, Ego A, Kaminiski M, Goffinet F, Zeitlin J (2014) Poor effectiveness of antenatal detection of fetal growth restriction and consequences for obstetric management and neonatal outcomes: a French national study. Int J Obstet Gynaecol 122(4):518–527. 10.1111/1471-0528.13148 10.1111/1471-0528.13148 25346493

[11] Chauhan SP, Beydoun H, Chang E, Sandlin AT, Dahlke JD, Igwe E, Magann EF, Anderson KR, Abuhamad AZ, Ananth CV (2014) Prenatal detection of fetal growth restriction in newborns classified as small for gestational age: correlates and risk of neonatal morbidity. Am J Perinatol 31(3):187–194. 10.1055/s-0033-1343771 23592315 10.1055/s-0033-1343771

[12] Gabbay-Benziv R, Aviram A, Hadar E, Chen R, Bardin R, Wiznitzer A, Yogev Y (2016) Pregnancy outcome after false diagnosis of fetal growth restriction. J Matern Fetal Neonatal Med 30(16):1916–1919. 10.1080/14767058.2016.1232383 27650628 10.1080/14767058.2016.1232383

[13] Dollberg S, Haklai Z, Mimouni FB, Gorfein I, Gordon ES (2005) Birth weight standards in the live-born population in Israel. Isr Med Assoc J 7(5):311–314 15909464

[14] Ringa V, Carrat F, Blondel B, Bréart G (1993) Consequences of misdiagnosis of intrauterine growth retardation for preterm elective cesarean section. Fetal Diagn Ther 8(5):325–330. 10.1159/000263847 8267867 10.1159/000263847

[15] Inder TE, Volpe JJ, Anderson PJ (2023) Defining the neurologic consequences of preterm birth. N Engl J Med 389(5):441–453. 10.1056/nejmra2303347 37530825 10.1056/NEJMra2303347

[16] Ben-Shmuel A, Sheiner E, Tsumi E, Wainstock T, Feinblum D, Walfisch A (2022) Early-term deliveries and long-term pediatric ophthalmic morbidity of the offspring. Int J Gynaecol Obstet 157(3):640–646. 10.1002/ijgo.13875 34383310 10.1002/ijgo.13875

[17] Gutvirtz G, Wainstock T, Sheiner E, Landau D, Walfisch A (2018) Pediatric cardiovascular morbidity of the early term newborn. J Pediatr 194:81-86.e2. 10.1016/j.jpeds.2017.09.060 29129352 10.1016/j.jpeds.2017.09.060

[18] Paz Levy D, Sheiner E, Wainstock T, Sergienko R, Landau D, Walfisch A (2017) Evidence that children born at early term (37–38 6/7 weeks) are at increased risk for diabetes and obesity-related disorders. Am J Obstet Gynecol 217(5):588.e1-588.e11. 10.1016/j.ajog.2017.07.015 28729012 10.1016/j.ajog.2017.07.015

[19] Walfisch A, Beharier O, Wainstock T, Sergienko R, Landau D, Sheiner E (2016) Early-term deliveries as an independent risk factor for long-term respiratory morbidity of the offspring. Pediatr Pulmonol 52(2):198–204. 10.1002/ppul.23529 27458900 10.1002/ppul.23529

[20] Hirata K, Ueda K, Wada K, Ikehara S, Tanigawa K, Kimura T, Ozono K, Sobue T, Iso H (2023) Neurodevelopmental outcomes at age 3 years after moderate preterm, late preterm and early term birth: the Japan environment and children’s study. Arch Dis Child Fetal Neonatal Ed. 10.1136/archdischild-2023-325600 10.1136/archdischild-2023-325600 37709498

[21] Baumfeld Y, Sheiner E, Wainstock T, Segal I, Sergienko R, Landau D, Walfisch A (2018) Elective cesarean delivery at term and the long-term risk for neurological morbidity of the offspring. Am J Perinatol 35(11):1038–1043. 10.1055/s-0038-1637001 29510422 10.1055/s-0038-1637001

[22] ACOG (2019) ACOG practice bulletin no. 204: fetal growth restriction. Obstet Gynecol 133(2):e97–e109. 10.1097/aog.0000000000003070 30681542 10.1097/AOG.0000000000003070

[23] Gaillard R, de Ridder MA, Verburg BO, Witteman JC, Mackenbach JP, Moll HA, Hofman A, Steegers EA, Jaddoe VW (2011) Individually customised fetal weight charts derived from ultrasound measurements: the generation R study. Eur J Epidemiol 26(12):919–926. 10.1007/s10654-011-9629-7 22083366 10.1007/s10654-011-9629-7 PMC3253277

[24] Al-Hafez L, Chauhan SP, Riegel M, Balogun OA, Hammad IA, Berghella V (2020) Routine third-trimester ultrasound in low-risk pregnancies and perinatal death: a systematic review and meta-analysis. Am J Obstet Gynecol MFM 2(4):100242. 10.1016/j.ajogmf.2020.100242 33345941 10.1016/j.ajogmf.2020.100242

